# Numerical Investigation into the Gas Production from Hydrate Deposit under Various Thermal Stimulation Modes in a Multi-Well System in Qilian Mountain

**DOI:** 10.3390/e23070800

**Published:** 2021-06-23

**Authors:** Bo Li, Yuan Ye, Tingting Zhang, Qingcui Wan

**Affiliations:** 1 State Key Laboratory of Coal Mine Disaster Dynamics and Control, Chongqing University, Chongqing 400044, China; 20192002022t@cqu.edu.cn (Y.Y.); 202020021033@cqu.edu.cn (T.Z.); 201920131062@cqu.edu.cn (Q.W.); 2 School of Resources and Safety Engineering, Chongqing University, Chongqing 400044, China

**Keywords:** gas hydrate, horizontal well, thermal stimulation, efficiency, numerical simulation

## Abstract

The primary objective of this study was to investigate the energy recovery performance of the permafrost hydrate deposit in the Qilian Mountain at site DK-2 using depressurization combined with thermal injection by the approach of numerical simulation. A novel multi-well system with five horizontal wells was applied for large-scale hydrate mining. The external heat is provided by means of water injection, wellbore heating, or the combinations of them through the central horizontal well, while the fluids are extracted outside from the other four production wells under constant depressurization conditions. The injected water can carry the heat into the hydrate deposit with a faster rate by thermal convection regime, while it also raises the local pressure obviously, which results in a strong prohibition effect on hydrate decomposition in the region close to the central well. The water production rate is always controllable when using the multi-well system. No gas seepage is observed in the reservoir due to the resistance of the undissociated hydrate. Compared with hot water injection, the electric heating combined with normal temperature water flooding basically shows the same promotion effect on gas recovery. Although the hydrate regeneration is more severe in the case of pure electric heating, the external heat can be more efficiently assimilated by gas hydrate, and the efficiency of gas production is best compared with the cases involving water injection. Thus, pure wellbore heating without water injection would be more suitable for hydrate development in deposits characterized by low-permeability conditions.

## 1. Introduction

**Figure 1:**
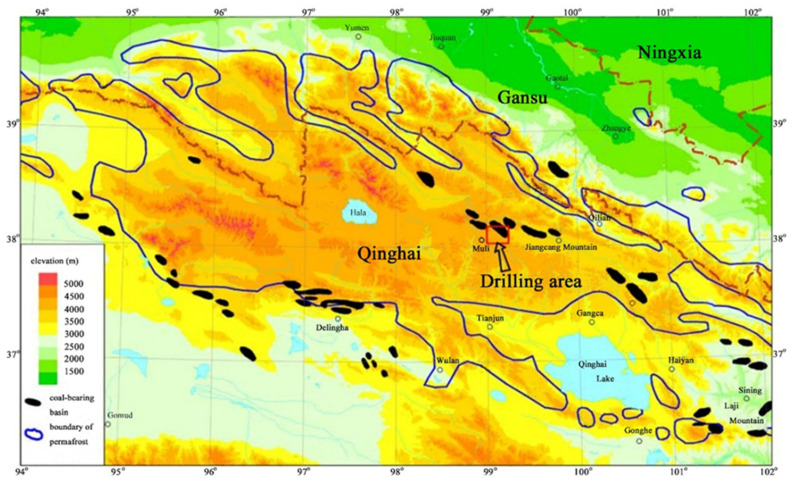
Location of the drilling area in the permafrost of Qilian Mountain.

With the rising desire for new energy and solving the global problem of climate change, natural gas hydrate has received more and more attention as the most promising energy source in recent years. Natural gas hydrate (NGH) is an ice-like compound generated by the combination of water and small gas molecules when they are situated at high pressure and low temperature conditions. Studies have shown that natural gas hydrates are largely preserved in frozen terrestrial areas and marine sediments [1]. It is characterized by high energy density, wide distribution range, large-scale, and shallow burial. As the Qilian Mountain permafrost in China has favorable formation conditions of natural gas hydrate, a drilling project was carried out in 2008–2009 with the purpose of hydrate exploration, and rock samples containing natural gas hydrate were resoundingly acquired [2]. Thereafter in 2011, the first hydrate mining test was carried out in the permafrost areas of Qilian Mountain by combining depressurization and thermal stimulation, and natural gas was successfully produced during the 101 h of hydrate mining experiment [3]. Qilian Mountain is situated in the Qinghai province of China, and it is also part of the Qinghai–Tibet Plateau Permafrost, as displayed in Figure 1 [4,5]. Four drilling holes (named as DK-1~DK-4) were completed in the areas with the possible occurrence of hydrates, and the field measuring data indicated that the pores and the fractures of the obtained cores are the main locations for the accumulation of gas hydrate [6]. The materials of the hydrate-bearing cores are mostly oil shale, sandstone, mudstone, and siltstone. It was found that natural gas hydrate is accumulated in the underground zones with a deepness of 133–396 m below the ground surface [7]. A study also found that the average temperature of the permafrost ground (*T*_0_) was in the range of −1 to −3 °C every year. The thermal gradient of the intrapermafrost areas *G*_1_ was about 0.011–0.033 °C/m, and the thermal gradient in the subpermafrost regions *G*_2_ was approximately 0.028–0.051 °C/m [8,9]. With these suitable geologic properties, large quantities of natural gas hydrate have been formed and stored in the permafrost areas of Qilian Mountain.

The main exploitation methods of natural gas hydrate contain the following items: depressurization [10,11,12,13], thermal stimulation [14,15,16], inhibitor injection [17,18], and gas replacement method (Such as CO_2_ and N_2_) [19,20]. According to its economic efficiency and energy utilization rate, depressurization is known as the most economically feasible method for field-scale mining of gas hydrate [11]. Because of the low-temperature environment in the permafrost zones of Qilian Mountain, the heat transfer rate and the available heat from the existing materials in the deposit are both extremely limited, leading to the restricted and undesirable gas extraction rate when using the depressurization strategy for hydrate dissociation at site DK-3 [21]. Subsequent studies indicate that desirable gas-to-water ratio and energy efficiency can be obtained under suitable injection and production conditions with the huff and puff method. This is because the huff and puff method has the advantages of enhancing the heat convection effect and reducing the heat absorption of the hydrate deposit [22,23]. To provide insightful guidance for field-scale exploitation of gas hydrate, people have employed various numerical codes to simulate the production potentials of various hydrate deposits. Grover et al. proposed a variety of cross-sectional models with the use of a single well, and then the simulation results were fully compared with various in-situ measuring data acquired from the Messoyakha field [24]. Moridis et al. [25,26,27] adopted the TOUGH + Millstone simulator (composed of two constituent codes: the TOUGH + HYDRATE and Millstone) for describing the flow, thermal, and chemical processes in hydrate-bearing media. Li et al. [28] analyzed the dynamic properties of gas hydrate development from a large hydrate simulator through numerical simulation. The results showed that most of the heat supplied from outside was assimilated by methane hydrate. Because the entropy production rate was not sensitive to the energy recovery rate under depressurization, the production pressure should be set as low as possible for the purpose of enhancing exploitation efficiency.

In addition to the production methods, the wellbore design is also an important factor affecting the overall exploitation efficiency. Feng et al. [29] numerically analyzed the hydrate mining behaviors in the area of the South China Sea by introducing varied horizontal well configurations. They confirmed that the energy acquisition rate of the dual wells would become much higher when they were located at the same horizontal plane. With the employment of a five-spot vertical well system, Wang et al. [16,30] studied the mining properties of gas hydrate with a variety of strategies in a cubic hydrate simulator. The effectiveness and the feasibility of such kind of multi-well system were confirmed for hydrate development. Recently, Liang et al. [31] numerically assessed the mining potential of the permafrost hydrates by adopting five horizontal wells at DK-2 of Qilian Mountain. It was indicated from the obtained results that favorable energy recovery could be acquired with appropriate heat injection and depressurization driving forces. Then Li et al. [32] investigated the gas production behaviors of methane hydrate dissociation induced by depressurization and electrical heating and proved the feasibility of the used method for hydrate exploitation in porous media. Besides, Liu et al. [33] proposed injecting water into a geothermal heat exchange well and achieved an increment cumulative gas production of 63.9%. However, the injected water flowing into the HBL also increases the formation pressure, which leads to a lower peak gas production rate at the early development stage. Li et al. [34] studied the dissociation behaviors of frozen methane hydrate in hydrate-ice-gas saturated porous media below freezing point with a single vertical well and found that the disturbance on gas and water in the pores could induce secondary hydrate formation. However, it is still not clear how the heat injection regimes (such as hot water injection, wellbore heating, and their combinations) will affect the energy recovery efficiency from the field-scale hydrate deposit using such a multi-well system.

The main objective of this work is to assess the gas recovery and hydrate mining potential from Qilian Mountain permafrost hydrate deposits at site DK-2 through depressurization combined with thermal injection by introducing a five-spot horizontal well system. We considered three heat injection approaches to figure out the most suitable thermal stimulation regime: electric heating, hot water injection, and electric heating combined with normal temperature water flooding. Various criteria, such as gas extraction rate and hydrate mining percentage, gas-to-water ratio, and energy ratio, were employed to evaluate the exploitation efficiency using the above production methods.

## 2. Production Strategy and Multiple Well Design

### 2.1. Exploitation Methods

Based on the field measurements, the temperature and pressure in the permafrost area of Qilian Mountain are considered to lie in the scope of 3.26–4.69 °C and 3.63–4.19 MPa, respectively. As the geographical conditions are characterized by low temperature, pure depressurization will be accompanied by a low recovery rate of natural gas due to the finite sensible heat stored in the underground deposit [21]. Many studies [15,16,23] came to a similar conclusion that the depressurization-induced gas production could be significantly improved if sufficient external heat is provided to overcome the problem of energy shortage. However, the adoption of pure thermal stimulation will also perform unfavorably if no other strategies are combined with it for hydrate decomposition. Thus, both depressurization and thermal stimulation were analyzed as the mining strategy.

The heat injection is accomplished with the following three modes: (1) electric heating, (2) hot water injection, and (3) electric heating in conjunction with normal temperature water flooding. For the case of electric heating, the hydrate deposit is heated by the wellbore under constant heating power *q*, and there is no mass injection all the time. For the hot water injection case, the same amount of heat is firstly consumed to raise the enthalpy of the injected water before it is supplied into the deposit. In the last case, operations of wellbore heating and water injection are conducted simultaneously, while the temperature of the externally provided water is the same as that of the hydrate deposit at the injection location. Then the thermal stimulation rate can be assured to be the same for the comparison of hydrate exploitation.

### 2.2. Five-Spot Well Design

**Figure 2:**
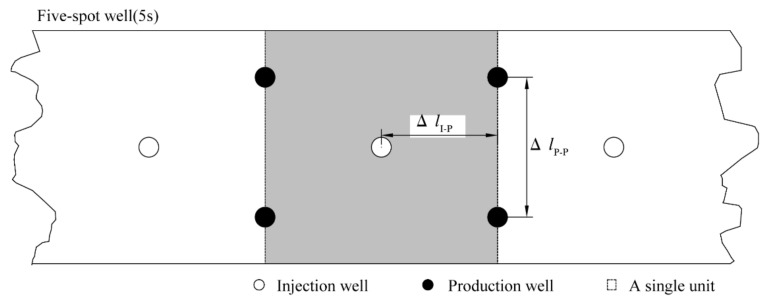
Illustration of the distribution pattern of the five-spot well system.

Figure 2 presents the distribution pattern of the five-spot well system (5S) which is used for the exploitation simulation of gas hydrate in the Qilian Mountain. The white wells are the heat injection wells, and the black wells are the production ones. All of these wells are placed in the hydrate-bearing layer horizontally. Due to the symmetrical properties of the system, we can only simulate the hydrate dissociation in a single rectangular zone, as plotted with the gray color in Figure 2. There are five working wells in each zone: an injection well at the central site of the strata for thermal stimulation and four production wells at the side borders of the zone for fluids extraction. The horizontal interval between the injection and the production wells in each simulated zone is Δ*l*_I-P_, while the vertical interval between the adjacent two production wells is Δ*l*_P-P_. When the hydrate mining is started, by electric heating or hot water injection, heat can be provided continuously from the center well to the hydrate-containing areas. At the same time, fluids (gas and water) are extracted out of the system from the other production wells by dropping their pressure to an invariable bottomhole pressure *P_W_*.

## 3. Numerical Models and Simulation Approach

### 3.1. Numerical Simulation Code

To perform the numerical simulation, the TOUGH + HYDRATE (T + H) simulator [35], which is a compositional code for the modeling of multi-phase and multi-component features related to hydrate transition processes in sophisticated geologic media, was employed as the basis of this study. Two representative models describing the equilibrium and the kinetic properties of hydrates are available in this code, and they were validated by Li et al. [36,37] via the laboratory results of two hydrate reactors. Besides, the equilibrium model is generally considered to act with better performance during predicting the physical and chemical features of various hydrate-associated processes based on the comparisons of the two submodels [11]. Therefore, the decomposition behaviors of gas hydrate are described by the equilibrium model in this numerical study.

### 3.2. Geometric Features and Domain Discretization Pattern

**Figure 3:**
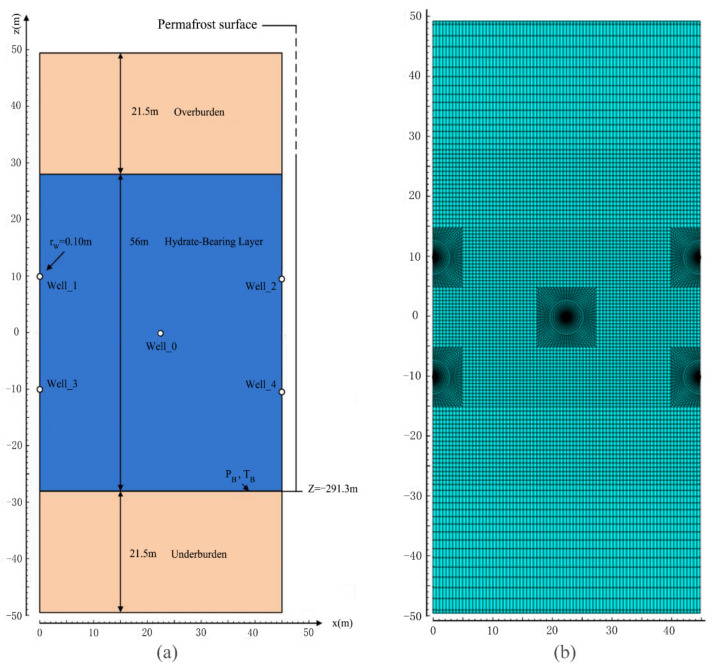
Schematic of (**a**) the five-spot well system and the geologic features of the domain, and (**b**) the adopted hybrid mesh for the simulation.

Based on the field measurement data and some published results, we obtained the simulation parameters as well as the physical features of the hydrate deposits chosen from the Qilian Mountain at the drilling location of DK-2, and some essential parameters are summarized in Table 1 [22,35,38]. The geometric features of the hydrate reservoir are displayed in Figure 3a, which illustrates the compositions of the entire concerned deposit containing the multiple horizontal well system. Because of the symmetry, only a single unit is considered in the simulation. Every unit is comprised of five horizontal wells with the radius of *r*_W_ = 0.10 m: one injection well set at the central point of the hydrate layer (Well_0) and four production wells placed at the sides of the domain (Well_1–Well_4). All the five wells are located in the interior area of the hydrate-bearing layer (HBL), and the locations of the upper and lower surfaces of the HBL are *z* = 28.0 m and *z* = −28.0 m, respectively. Nevertheless, only half of Well_1–Well_4 plays a role in recovering fluids from the concerned single unit. The precise locations of the five wells in the Cartesian coordinate system are as follows: Well_0 (22.5, 0), Well_1(0, 10), Well_2 (45, 10), Well_3 (0, −10), and Well_4 (45, −10). Therefore, the wellbore intervals shown in Figure 2 are Δ*l*_P-P_ = 20.0 m and Δ*l*_I-P_ = 22.5 m. In addition to the hydrate layer, another two layers (21.5 m thick) without the presence of hydrate were also involved in the simulated domain: the overburden layer (OB) and the underburden layer (UB), which are located in the regions of 28.0 ≤ *z* ≤ 49.5 m and −49.5 ≤ *z* ≤ −28.0 m, respectively. The physical properties of the two boundary layers are similar to that of the HBL. Although the horizontal wells are usually hundreds of meters long, the working conditions of the wells are assumed to be the same along their extension direction (the *y* axis). Then only a single unit with the domain thickness Δ*y* = 1 m needs to be considered because of symmetry along the *y* direction.

The aforementioned domain is discretized into a two-dimensional hybrid grid, of which the total number of elements is 13,540, as shown in Figure 3b. To obtain precise forecasting of energy recovery and to accurately obtain the seepage properties of the fluids in the pores and the evolutions of the various materials in the concerned domain, a fine discretization is adopted in the area of the HBL. Due to the drastic chemical and physical courses occurring in the vicinity of the five horizontal wells, the region within *r* < 3.0 m around every wellbore is represented by a rigid cylindrical mesh. These cylindrical elements are connected with the outside brick gridblocks by a series of radially graded mesh. In the process of numerical simulation, through dropping their pressures below the initial level, fluids can be extracted outside from four gridblocks located at the innermost sections of the wellbore (*r* = 0.1 m). The boundary elements, which are exactly located at the upper and lower surfaces of the deposit in Figure 3, are applied with invariable simulation conditions. Such discretization will result in 54,160 coupled equations which require to be computed synchronously when the hydrate dissociation is treated by the equilibrium mode without inhibitors.

### 3.3. Initialization of the System

The initial situations of the hydrate reservoir are obtained based on the initialization strategies depicted by Li et al. [22]. The thermodynamic states of the whole domain are determined by calculating the pressure and temperature using the following equations:

(1) $$P=\rho_{r}gh+\rho_{w}g\left(h-H\right)+P_{0}$$

(2) $$T=273.15+G_{2}\left(h-H\right)$$

where *ρ_r_* stands for the density of the rocks obtained in the hydrate deposit (2000 kg/m^3^) [9], *ρ_w_* represents the density of aqueous water stored in the reservoir (l000 kg/m^3^), *g* is the gravitational acceleration (9.81 m/s^2^), *h* and *H* are the depth of a specific element and the thickness of the intrapermafrost layer, respectively, and *P*_0_ is the atmospheric pressure (1.01 × 10^5^ Pa). The parameter *H* is determined as

(3) $$H=\left(273.15-T_{0}\right)/G_{1}$$

Table 1 also presents the thermophysical parameters of *G*_1_, *G*_2_, and *T*_0_, which are obtained according to the surveyed results in the literature [9]. In addition, the initial pressure and temperature of the elements at the base of the hydrate layer are determined to be *P_B_* = 4.19 MPa and *T_B_* = 277.84 K, respectively.

After initialization, the pressure of the system is very close to the equilibrium pressure, which is aimed to induce easy destabilization of gas hydrates under mild depressurization conditions [22]. The temperatures and pressures of the topmost and bottommost elements (the boundary sites) are calculated to be 275.76 K, 3.43 MPa and 278.51 K, 4.41 MPa, respectively, and they remain invariable during the entire simulation period. Furthermore, the phase saturations of aqueous water (*S_A_*) and hydrate (*S_H_*) in the HBL are assigned with the initial value of 0.60 and 0.40, respectively. It is assumed that there is no free gas in the pores of the OB and UB sections, which indicates that the water saturation is *S_A_* = 1.0. The thermodynamic conditions of the grid and the phase state of all the materials in the system will maintain stable if no interferences are introduced from the external environment.

## 4. Results and Discussion

### 4.1. Production Characteristics

The multi-well system involving five horizontal wells shown in Figure 3 was used to facilitate the hydrate mining from the DK-2 hydrate deposit in the permafrost of Qilian Mountain. Three combination methods involving thermal stimulation and depressurization were employed to enhance hydrate exploitation. In order to assure an easy heat and mass injection at Well_0, all the horizontal wells were depressurized for 100 days to release part of the pores which were previously occupied by hydrates near the wellbore before the thermal stimulation was started.

Then the central well (Well_0) was changed to act as an injector, which was operated to provide heat to the inner strata with a steady rate *q* through the injection of hot water or electrical heating. The externally supplied heat was aimed to restrain the possibility of production abortion due to flow blockage by ice formation or hydrate regeneration. At the same time, the other four production wells (Well_1–Well_4) were maintained to be situated under barometric pressure 0.101 MPa for continuous gas recovery. Such kind of treatment is similar to the production strategies reported in the literature [31]. Three cases with various heat injection modes were simulated: (1) electric heating (E); (2) hot water injection (W); (3) electric heating combined with normal temperature water flooding (E-W). The water injection rate in the two latter cases was set to be *V* = 5.25 × 10^−5^ kg/s. The heat consumption rates in all three cases were *q* = 40 W per unit length (1.0 m) of the well. The case employing hot water injection in the 5S system was specifically analyzed as the reference case (Ref). In addition, another two runs with higher heat injection rates (80 W and 160 W) using the hot water injection method were implemented to find out the dependence of gas production on heat injection rate.

#### 4.1.1. Gas Production

**Figure 4:**
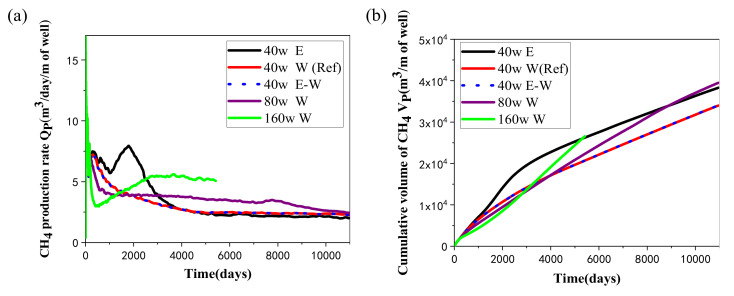
Change profiles of (**a**) the methane recovery rate *Q*_P_, and (**b**) the accumulated volume *V*_P_ of CH_4_ extracted by the five-spot well system under various heat injection modes.

The change rules of the gas recovery rate (*Q*_P_) and the total volume of the obtained methane (*V*_P_) under various heat injection rates and thermal stimulation methods in the hydrate deposit at DK-2 are presented in Figure 4. Figure 4a shows that *Q*_P_ firstly grows dramatically in the early depressurization stage (for 100 days), and then it declines to lower levels in the following period. In the reference case, the peak value of *Q*_P_ is approximately 26 m^3^/day per unit length of well in the concerned simulation area. Subsequently, due to the reduced heat utilization abilities of gas hydrate in the later mining process, *Q*_P_ tends to drop to a relatively low level. At the same time, it could be discovered from Figure 4 that the *Q*_P_ of the two methods, hot water injection and electric heating combined with normal temperature water flooding, are basically the same. This indicates that when the mass injection is introduced, different methods have no effect on the decomposition of hydrate. Moreover, it is clearly observed in Figure 4a that when hot water is injected, the rate of gas extraction is lower than that of electric heating, which indicates that direct electric heating at the wellbore is more advantageous than the situations when water injection is introduced. This is because the seepage ability of water is strongly restricted by the low permeability of the deposit.

It is shown in Figure 4b that *V*_P_ tends to rise consecutively in the entire mining period with different methods, while its rising speed in the initial stage is distinctly faster than that in the later period. Such change properties are in conformity with the various behaviors of the *Q*_P_ curves discussed in Figure 4a. In the reference case, the accumulated volume of the produced CH_4_ is about 3.40 × 10^4^ ST m^3^ in 30 years. In addition, compared with electric heating, the gas production of the cases involving water injection is a little lower than that with pure electric heating. Although the injected water may be able to carry the heat into the hydrate undissociated area with a faster rate by thermal convection regime, it will also raise the local pressure of the HBL due to the limited space of the porous media, making the equilibrium temperature at which gas hydrate can be dissociated rise to a higher level. In other words, the negative effect of pressure increase is more obvious than the positive effect of thermal convection, which makes it more difficult for gas hydrate dissociation under water injection. Thus, pure wellbore heating without water injection would be more suitable for the commercial development of gas hydrate in hydrate deposits characterized by low-permeability conditions.

#### 4.1.2. Rates of Water Production and Hydrate Decomposition

**Figure 5:**
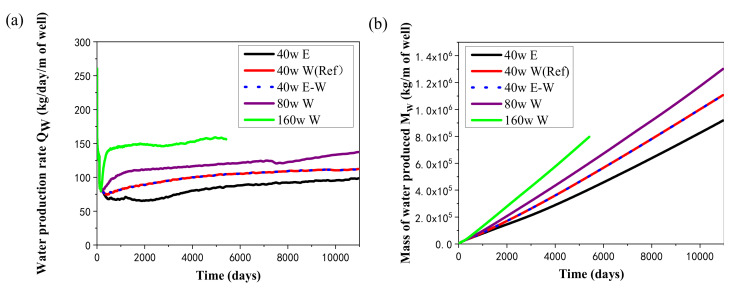
Change profiles of (**a**) the water extraction rate *Q*_W_, and (**b**) the accumulated mass of extracted water *M*_W_ in the five-spot well system using different injection modes.

Figure 5 shows the change tendency of the water extraction rate (*Q*_W_) and the total mass of water released from the system (*M*_W_) under different heat injection rates and thermal stimulation patterns in the hydrate deposit at DK-2. For all the cases with water injection, *Q*_W_ increases rapidly in a short period of time, which is resulted from the external water mass injection. The compression effect due to water invasion causes a larger pressure difference between the deposit and the production wells, which is also responsible for the faster water seepage rate in the system. In the reference case, *Q*_W_ increases at first and then decreases quickly in the first 100-days depressurization stage. Then it descends sharply to about 74 kg/day per unit length of well at about 400 days when the free water in the pores near the production wells has been nearly exhausted. Generally, the water releasing rate from the well is controllable when using the 5S system for the exploitation of permafrost gas hydrates.

As the variation tendency of *Q*_W_ is not violent during most of the mining period in Figure 5a, *M*_W_ almost rises in a linear manner in the entire energy recovery period, as displayed in Figure 5b. The cumulative mass of the extracted water reaches about 1.11 × 10^6^ kg (=1110 tons) when the production operation is ended for each single simulation domain in the reference case. The average water extraction speed is 112.47 kg/day/m of the well, which is in accordance with the plotted profiles in Figure 5a and is manageable with current industrial technologies. It can also be seen from Figure 5 that different injection rates with hot water have a great impact on the water production rate, and both *Q*_W_ and *M*_W_ have obvious changes, which means that the water released from the domain is sensitive to the amount of hot water injection in the 5S system.

**Figure 6:**
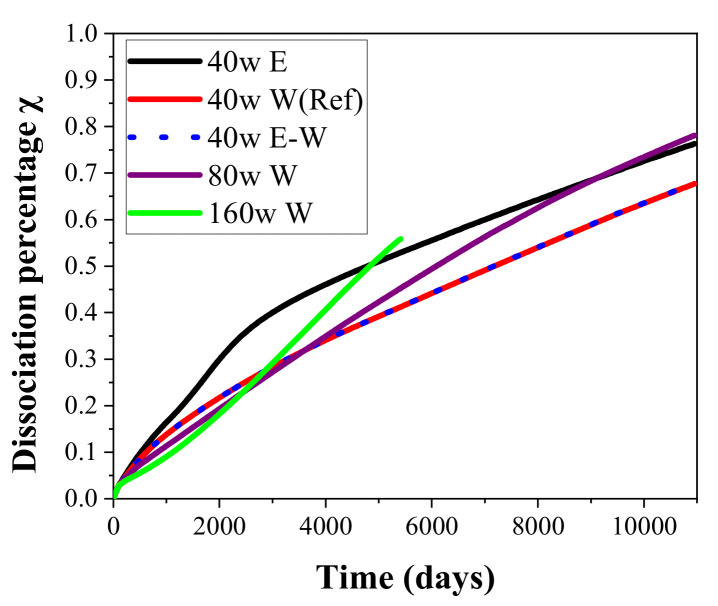
Profiles of the decomposition percentage of hydrate *χ* during its mining by the five-spot well system using various heat injection modes.

The hydrate decomposition percentage *χ*, which means the ratio of the decomposed hydrate to the initial hydrate presented in the deposit, was employed as another indicator to assess the effectiveness of the employed exploitation methods. Figure 6 illustrates the evolution of the *χ* during hydrate mining under different heat injection methods using the 5S systems in the hydrate deposit at DK-2. It can be seen that the change tendency of dissociation percentage is similar to that of the obtained *V*_P_ curve in each case presented in Figure 4b, indicating that the majority of the gas produced is extracted from the dissociation reaction of gas hydrate. In the reference case, *χ* rises to about 66.7% when the production process is terminated. In the first 10 years, we can see that it reduces with the increase of *q*, while in the following 20 years, it increases with the rise of *q* because of the further enhanced promotion effect of heat injection on hydrate decomposition.

#### 4.1.3. Gas-to-Water Ratio and Energy Ratio

**Figure 7:**
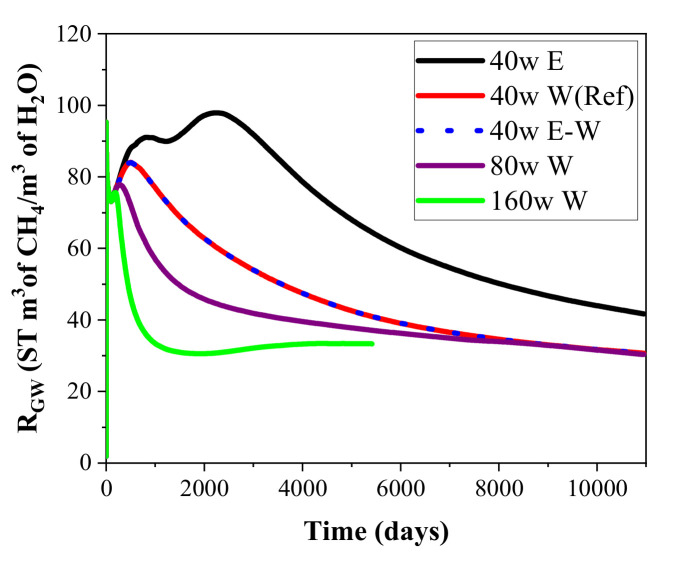
Change profiles of the gas-to-water ratio *R*_GW_ during hydrate mining by the five-spot well system under different heat injection modes.

The change profiles of the gas-to-water ratio *R*_GW_ during energy recovery from the hydrate deposit at DK-2 using the five-spot well system with different heat supply rates and thermal stimulation methods are displayed in Figure 7. The gas-to-water ratio is calculated by

(4) $$R_{\mathrm{GW}}=V_{P}/V_{W}$$

where *V*_W_ represents the volume of the extracted water under standard state (m^3^). *R*_GW_ is often employed as another optional indicator to evaluate the exploitation efficiency of the adopted mining technology. It is shown in Figure 7 that *R*_GW_ rises continually to the highest level of 84 in about 1300 days in the reference case. This is because the water seepage in the pores is largely suppressed by the fast flow of gas in this period, as mentioned in Figure 4a and Figure 5a. Subsequently, *R*_GW_ presents the tendency of declining with time because of the weakened gas recovery rate and the enhanced water extraction rate. Eventually, the *R*_GW_ will drop below 30. As there is no water injection in the case of pure electric heating, *R*_GW_ is always higher than the other runs due to the smaller amount of water extraction shown in Figure 5.

**Figure 8:**
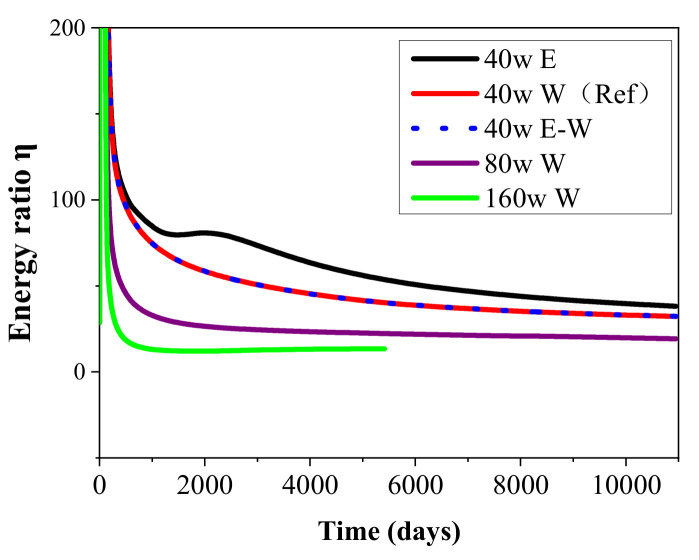
Change profiles of the energy ratio *η* in the five-spot well system under different heat injection modes.

Figure 8 shows the change tendency of the energy ratio *η* during energy recovery from the DK-2 hydrate deposit using the five-spot well system under different heat injection rates and thermal stimulation methods. The definition of energy ratio can be made as to the ratio of the obtained energy through the combustion of the recovered CH_4_ to the total energy cost during the mining process. The expended energy is composed of two items: the injected heat *Q* and the mechanical energy for the pump *W*. Thus, *η* can be calculated as follows:

(5) $$\eta =\Delta H_{C}/\left(Q+W\right)$$

where Δ*H_C_* is the enthalpy of methane combustion (889.6 kJ/mol at 1 atm and 25 °C). The detailed determination of the above energy sources can be obtained from previous studies [22]. It supplies one more insightful criterion for the evaluation of the overall mining efficiency during the heat injection-stimulated dissociation of gas hydrate.

It is shown in Figure 8 that *η* reduces persistently to the minimum level of about 30% in the period when the recovery of methane is sustained in the reference case. Such decline mainly results from the decreased hydrate mining and gas extraction rate due to the gradually raised heat transfer resistance from Well_0 to the undissociated hydrate regions. In addition, the energy ratios in the cases with water injection are nearly the same with each other all the time, which is caused by the similar gas production profiles discussed in Figure 4.

### 4.2. Comparison of Hot Water Injection

In this study, another two runs with a higher thermal stimulation rate (80 W and 160W) in the form of hot water injection were also conducted for gas recovery from the permafrost deposit at DK-2. The temperature of the pumped water of the two cases remains the same as that of the reference case, while the water supply rates are raised to 1.05 × 10^−4^ kg/s and 2.10 × 10^−4^ kg/s, respectively. The simulations are performed in the same domain, and the obtained results of the two runs are also plotted in Figure 4, Figure 5, Figure 6, Figure 7 and Figure 8. With the tremendous secondary hydrate formation under higher water injection rate, the generated blocking impact on the seepage abilities of gas and water between the injection and production wells becomes more severe, and the simulation process in the case of *q* = 160 W is broken off at about *t* = 5400 days.

One can see from Figure 4 and Figure 6 that *Q*_P_, *V*_P_, and *χ* of the 80 W and 160 W cases are all markedly smaller than those of 40 W in the first 10 years, which means that more water injection will unexpectedly obtain a slower production process at the beginning. This is caused by the more significant increase in the pore pressure under a higher mass injection rate, which further results in unfavorable dissociation conditions for gas hydrate. On the other hand, the situation has changed a great deal since then, as the gas extraction and hydrate mining rates are both increased to be larger than those of the reference case. Therefore, the positive effect of a higher heat stimulation rate on the hydrate mining process will be delayed using the 5S system with the hot water injection method.

Figure 5 shows that the releasing rates of water in the two cases with higher injection speeds are both located at a relatively higher level. The higher pressure caused by the larger mass injection rate plays a role in promoting the seepage rate of water from Well_0 to the other production wells. Then the *R*_GW_ also gradually reduces to a lower situation than the case of 40 W in Figure 7 due to the increase in water produced from the wells. On the other hand, the energy ratio is observed to drop notably on raising the heat injection rate from 40 to 160 W in the three runs. Such decline means that the heat utilization efficiency of gas hydrate generally reduces with the rise of heat injection rate, which further results in a lower energy ratio. Taking into account the effect of different heat injection methods on *V*_P_ and *R*_GW_ mentioned previously, the operation methods of heat injection need to be chosen based on the actual geological conditions of the deposit to ensure a successive and desirable gas recovery process.

### 4.3. Spatial Distributions

#### 4.3.1. Spatial Distributions of S_H_ and S_G_

**Figure 9:**
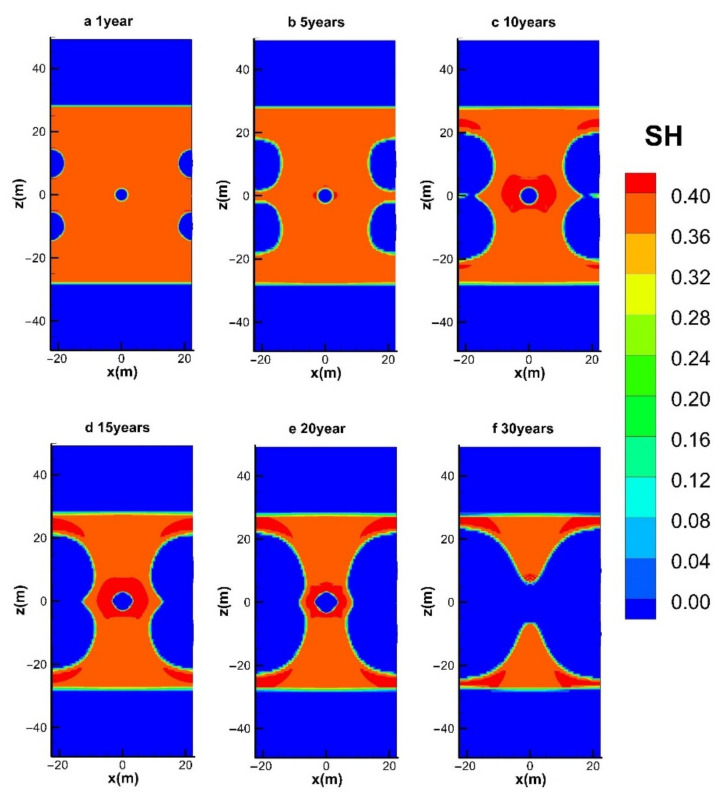
Variation in the spatial distribution of *S*_H_ with hot water injection in the five-spot well system.

**Figure 10:**
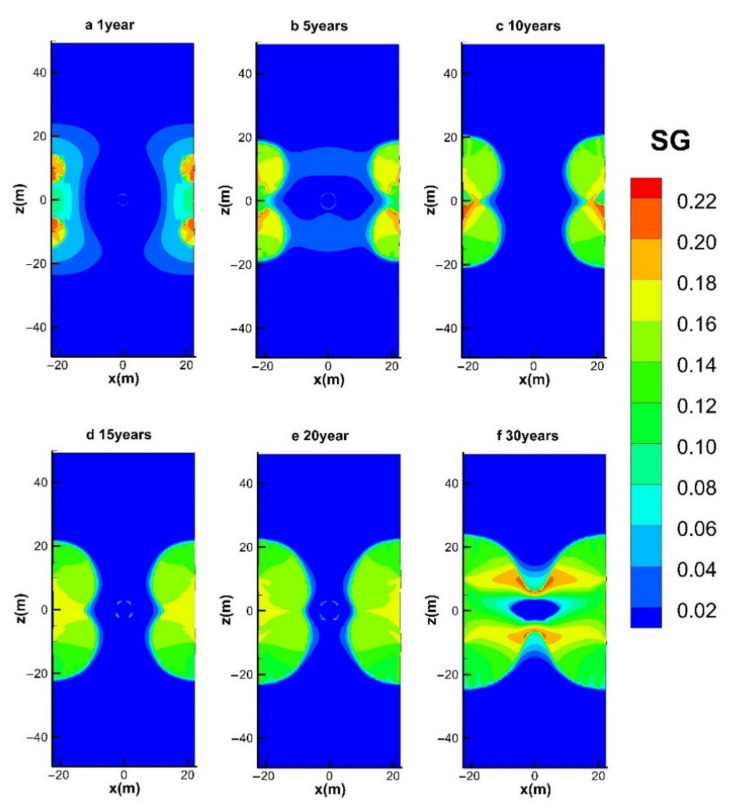
Variation in the spatial distribution of *S*_G_ with hot water injection in the five-spot well system.

The evolutions of the spatial distributions of hydrate (*S*_H_) and gas (*S*_G_) during the entire mining process in the reference case (hot water injection with *q* = 40 W) are shown in Figure 9 and Figure 10, respectively. The time points selected for the six figures are at *t* = 1, 5, 10, 15, 20, and 30 years, respectively. The chart includes the condition of the entire hydrate deposit presented in Figure 3a.

It is shown in Figure 9a that the hydrate near the five wells dissociated earlier, and the decomposition reaction mainly takes place at five cylindrical interfaces around the wells. The hydrates at the four production wells seem to decompose faster, which implies a more significant influence on hydrate dissociation imposed by the depressurized conditions. It shows that the hydrate reformation around the central injection well is not obvious, which is because the injected hot water pushes the hot fluids generated near the center well to the surrounding area and provides heat to inhibit the formation of secondary hydrates. However, the amount of secondary hydrate formation can be only limited under relatively low water injection conditions. Once the thermal stimulation rate increases from 80 W to 160 W by raising the water supply rate from 1.05 × 10^−4^ kg/s and 2.10 × 10^−4^ kg/s, the pressure near the injection well will be raised to a much higher level, which causes more favorable formation conditions for the secondary hydrate. Then the flow channels between the injection well and the production wells will be severely blocked. That is why the simulated process in the case of *q* = 160 W is unexpectedly broken off at about 5400 days, as mentioned in Figure 4, Figure 5, Figure 6, Figure 7 and Figure 8. The dissociation surfaces originated from the side wells gradually enlarge towards Well_0, and then these surfaces are eventually linked with each other after *t* = 10 years, as depicted in Figure 9b,c. It is noted from Figure 9a–e that the expansion rate of the dissociation interface near Well_0 is extremely slow during the whole water injection process, which implies the forceful prohibition influence of the pressure increase on the hydrate decomposition in this region. The injected heat can only take effect when the hot water successfully penetrates the hydrate undissociated area near the four production wells. There are still a lot of hydrates retained in the deposit at the end of the mining stage, as presented in Figure 9f.

Figure 10a illustrates that the part of the gas released from hydrates is trapped in the vicinity of the four production wells, and all the pores near Well_0 were totally occupied by the injected water. When the dissociation interfaces further expand to the inner area, the accumulation of gas becomes more obvious due to the disappearance of solid hydrates. In the following period (Figure 10c–e), the gas saturation near the injection well nearly always remains 0. It further proves that the injected hot water does not make immediate dissociation of gas hydrate due to the raised pressure conditions in this area. Therefore, the produced gas in Figure 4 is mostly obtained under the effect of depressurization from the four production wells. After all the dissociation interfaces are connected with the central well (Figure 9f), the injected water could diffuse more easily to the hydrate zone, leading to an enlarged gas-saturated area. Moreover, no gas seepage is observed to appear in the OB and UB as the undissociated and regenerated hydrate could hinder the transportation of fluids by dropping the effective permeability obviously in the reservoir.

#### 4.3.2. Spatial Distribution of T

**Figure 11:**
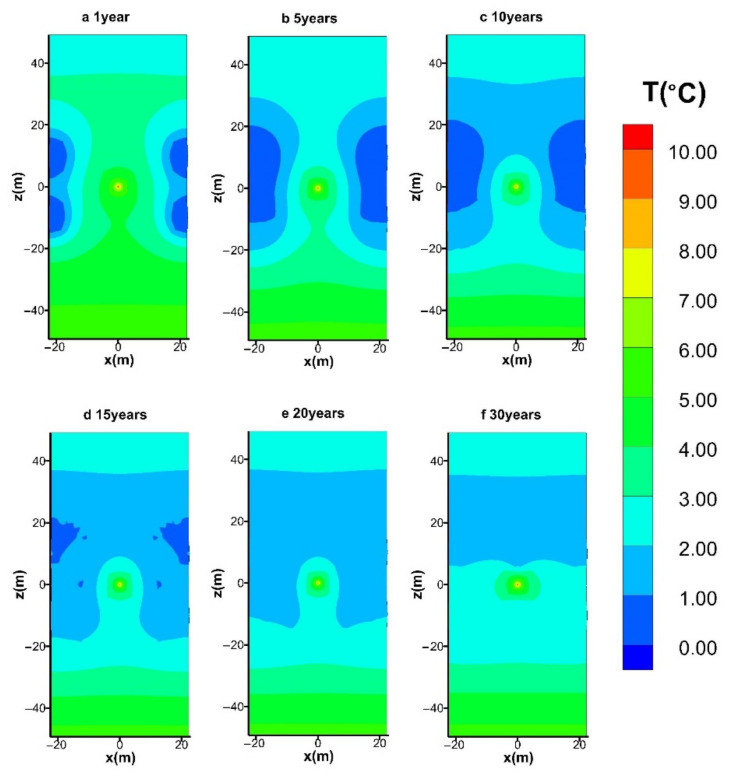
Variation in the spatial distribution of *T* with hot water injection in the five-spot well system.

The variation in the spatial temperature distribution during the entire mining course in the reference case (hot water injection with *q* = 40 W) is shown in Figure 11. The following features can be noticed from this figure: (i) the temperatures close to the producing wells decline significantly as a result of the enthalpy consumption by the phase transition of hydrates, as shown in Figure 11a; (ii) the supplied heat could only raise the temperature of the strata slightly, and most of the injected water is trapped surrounding Well_0, which results in relatively slow heat diffusion rate from the central well to the hydrate areas; (iii) the low-*T* regions (*T* < 1.0 °C) gradually contract with time when the injected water has the ability to flow to these locations under the pressure difference (Figure 11a–f); (iv) the temperature of the majority of the domain is always situated below 10 °C, which infers that the amount of lost heat during the water injection process is relatively limited.

#### 4.3.3. Spatial Distribution of S_I_

**Figure 12:**
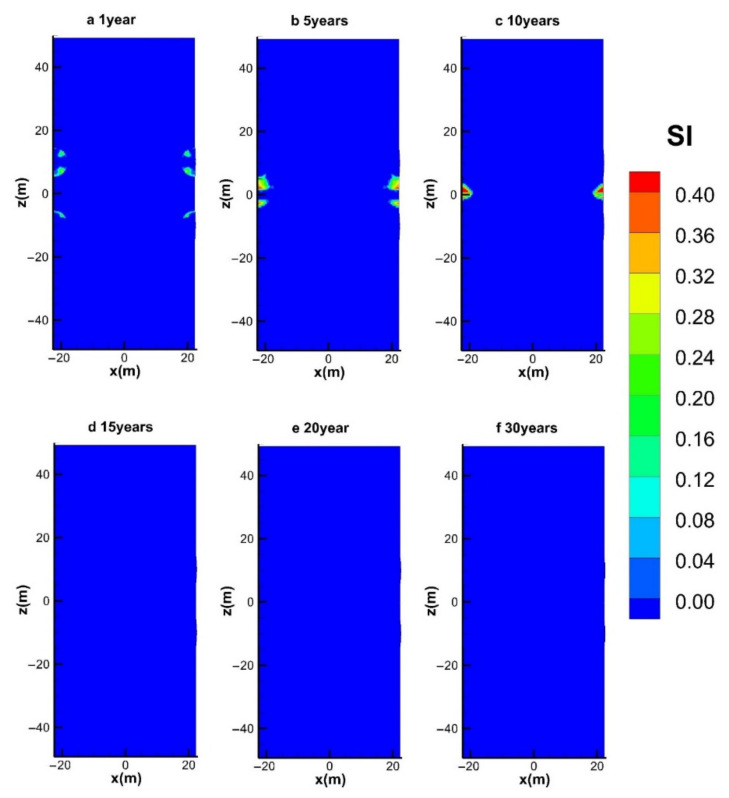
Variation in the spatial distribution of *S*_I_ with hot water injection in the five-spot well system.

Figure 12 displays the variation in the spatial distribution of ice (*S*_I_) in the deposit during the entire mining course in the reference case. As the sensible heat consumption of the system is the main source for hydrate dissociation under sharp depressurization in the initial exploitation period (Figure 12a), a small quantity of ice is formed near the production wells. The transformation of aqueous water into solid ice could provide a certain amount of latent heat for promoting hydrate dissociation [39]. Figure 12b shows that the ice transition phenomenon becomes more severe before the injected hot water can be effectively transferred to these zones. After that, the formed ice gradually diminishes and finally disappears under the heating effect of the injected hot water (Figure 12c–f). Therefore, the externally provided heat could exclude the possibility of blockage of the flow channels between the injection and production wells.

#### 4.3.4. Spatial Distribution of P

**Figure 13:**
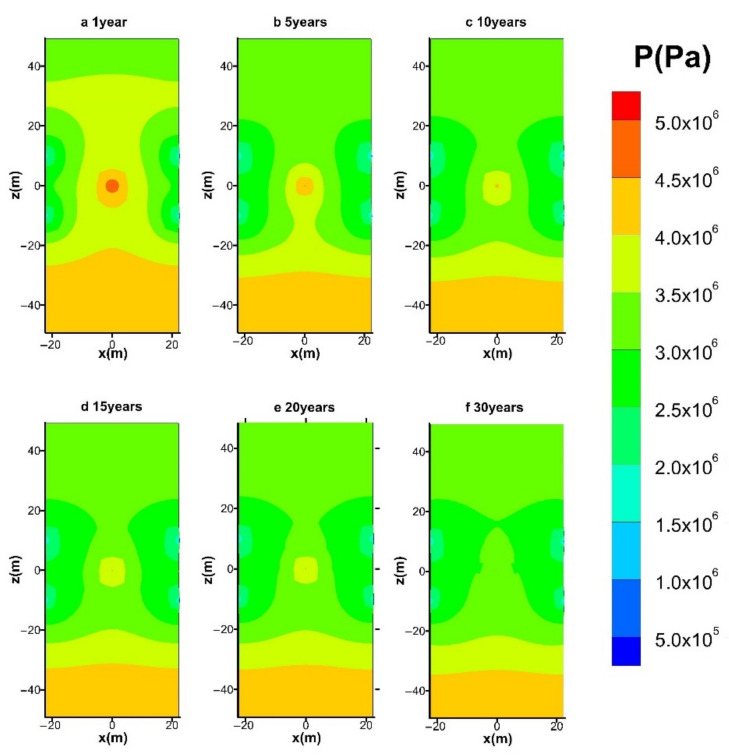
Variation in the spatial distribution of *P* with hot water injection in the five-spot well system.

Figure 13 shows the variation in the spatial distribution of *P* in the course of the 30-years mining process in the reference case (hot water injection with *q* = 40 W). Figure 13a displays that the pressure in the region surrounding the injection well is raised to a level that is even higher than that of the overburden layer. Such pressure increase results from the water accumulation when the fluids transportation ability of the deposit is still unfavorable for efficient water seepage. When the dissociation surfaces of the four side wells extend to the center well, the flow blockage between them will be partly relieved (Figure 13b), and the high-pressure zones gradually faded away with the continuous release of water. Moreover, the variation in the pressure distribution becomes relatively gentle in the following production process (Figure 13c–f). This means that the hydrate mining process is relatively stable and sustainable.

### 4.4. Effect of Injection Modes on Hydrate Dissociation

**Figure 14:**
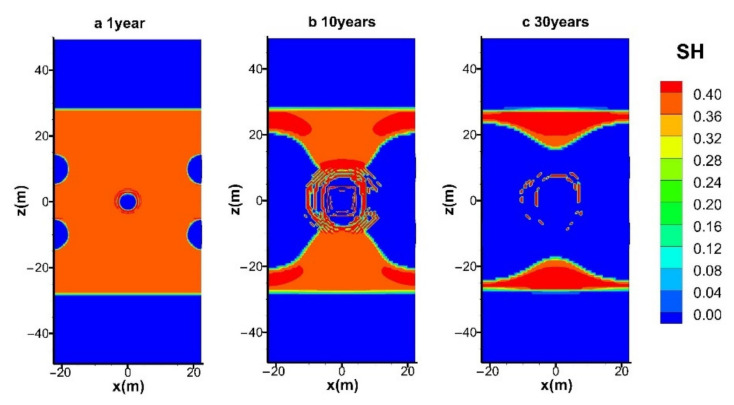
Variation in the spatial distribution of *S*_H_ with electric heating in the five-spot well system.

**Figure 15:**
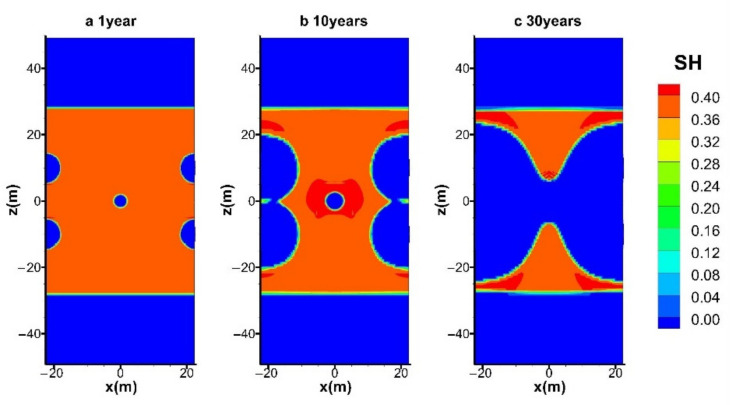
Variation in the spatial distribution of *S*_H_ with electric heating and normal temperature water flooding in the five-spot well system.

Figure 14 and Figure 15 show the spatial distribution of *S_H_* with electric heating and electric heating combined with normal temperature water flooding during gas production at site DK-2. Both cases have the same thermal injection rate of *q* = 40 W. The difference caused by different heat injection modes is not remarkable in the early period, as depicted in Figure 14a and Figure 15a. We can see that the phenomenon of hydrate regeneration is more obvious at *t* = 10 years when using the electric heating method, and the location of secondary hydrate revolves around the central well (Figure 14b), which acts as a barrier to inhibit subsequent production.

On the contrary, the formation of secondary hydrate can be more favorably controlled by water injection (Figure 9c and Figure 15b). This is because the injected heat could be carried to the inner hydrate zones more effectively under the effect of thermal convection during water flooding. In addition, it also shows that the spatial distribution of *S_H_* with hot water injection and electric heating combined with normal temperature water flooding is nearly the same, which is in accordance with the results discussed in Figure 4, Figure 5, Figure 6, Figure 7 and Figure 8. In general, under current technology, hot water injection has the ability to reduce the risk of secondary hydrate formation, while pure electric heating could facilitate gas recovery a little faster.

## 5. Conclusions

In this numerical study, the mining of the DK-2 permafrost hydrates in the Qilian Mountain was conducted in a multi-well system using depressurization and different heat injection modes. According to the simulated results, the following conclusions can be obtained:

1. Gas recovery behaviors of hot water injection and electric heating combined with normal temperature water flooding are basically the same. When the mass injection is introduced, different methods have no obvious effect on the decomposition of hydrate under the condition of the same heat injection rates;
2. When the water injection is applied, the seepage ability of water is strongly constrained by the low permeability of the deposit. Most of the injected water is trapped surrounding the central well. The negative effect of pressure increase is more obvious than the positive effect of thermal convection, which makes it more difficult for hydrate dissociation in the early water injection period;
3. The positive effect of a higher heat stimulation rate on the hydrate mining process will be delayed using the 5S system with the hot water injection method. The pumped hot water has the ability to reduce the risk of secondary hydrate formation under a suitable injection rate, while pure electric heating could facilitate the gas recovery a little faster;
4. The expansion rate of the dissociation interface near the injection well is extremely slow during the whole water injection process. The produced gas is mostly obtained with the promotion of pressure reduction from the four production wells. The injected heat can only take effect when the hot water successfully penetrates the hydrate undissociated area near the four production wells;
5. Part of the gas released from hydrates is trapped in the vicinity of the four production wells. The accumulation of gas becomes more obvious with the disappearance of solid hydrates. No gas seepage is observed to occur in the OB and UB sections as the undissociated and the regenerated hydrate could hinder the transportation of fluids by dropping the effective permeability obviously in the reservoir;
6. A small quantity of ice is formed near the production wells due to enthalpy consumption of the system by gas hydrate. It gradually diminishes and finally disappears under the heating effect of the injected hot water. The externally provided heat could exclude the possibility of blockage of the flow channels between the injection and production wells.

## Figures and Tables

**Table 1:** Physical features and simulation parameters of the permafrost hydrate deposit.

Parameter	Value
Thickness of hydrate-bearing area	56.0 m
Thickness of boundary layers	21.5 m
Depth of HBL underground	235 m
Interval of injection and producing wells Δ*l*_I-P_	22.5 m
Interval of two producing wells Δ*l*_P-P_	20.0 m
Gas composition	100%CH_4_
Initial phase saturations in the HBL	*S*_H_ = 0.40, *S*_A_ = 0.60
Ground temperature of permafrost surface	*T*_0_ = 271.56 K
Thermal gradient of intrapermafrost region	*G*_1_ = 0.013 °C·m^−^^1^
Thermal gradient of subpermafrost region	*G*_2_ = 0.028 °C·m^−^^1^
Intrinsic permeability of the domain	*k* = 1 mD
Porosity of the media Φ	0.30
Composite thermal conductivity model [35]	$k_{\Theta C}=k_{\Theta \mathrm{RD}}+\left(S_{A}{}^{1/2}+S_{H}{}^{1/2}\right)\left(k_{\Theta \mathrm{RW}}-k_{\Theta \mathrm{RD}}\right)+\Phi S_{I}k_{\Theta I}$
Thermal conductivity of dry porous media *k_ΘRD_*	1.0 W/(m K)
Thermal conductivity of saturated water porous media *k_ΘRW_*	3.1 W/(m K)
Capillary pressure model [38]	$P_{\mathrm{cap}}=-P_{01}{\left({\left(S^{*}\right)}^{-1/\lambda }-1\right)}^{1-\lambda }$
	$S^{*}=\left(S_{A}+S_{\mathrm{irA}}\right)/\left(1-S_{\mathrm{irA}}\right)$
*S_irA_*	0.29 [22]
Capillary equation exponent *λ*	0.45
Capillary pressure without solid phase *P*_01_	10^5^ Pa
Relative permeability Model [35]	$k_{\mathrm{rA}}={\left(S_{A}^{*}\right)}^{n}$
	$k_{\mathrm{rG}}={\left(S_{G}^{*}\right)}^{nG}$
	$S_{A}^{*}=\left(S_{A}-S_{\mathrm{irA}}\right)/\left(1-S_{\mathrm{irA}}\right)$
	$S_{G}^{*}=\left(S_{G}-S_{\mathrm{irG}}\right)/\left(1-S_{\mathrm{irA}}\right)$
Permeability reduction exponent *n*	3.572 [22]
Gas permeability reduction exponent *n_G_*	3.572
*S_irG_*	0.05
*S_irA_*	0.30

## Nomenclature

*G*_1_	thermal gradient within the frozen layer (°C/m)
*G*_2_	thermal gradient below the frozen layer (°C/m)
*H*	permafrost thickness (m)
*k*	intrinsic permeability (m^2^)
*k*_rA_	aqueous relative permeability (m^2^)
*k*_rG_	gas relative permeability (m^2^)
*k*_ΘC_	thermal conductivity (W/(m·K))
*k*_ΘRD_	thermal conductivity of dry porous medium (W/(m·K))
*k*_ΘRW_	thermal conductivity of fully saturated porous medium (W(m·K))
*k*_ΘI_	thermal conductivity of ice (W/(m·K))
*M*_W_	cumulative mass of produced water (kg)
*P*	pressure (Pa)
*P*_B_	initial pressure at base of HBL (Pa)
*P*_W_	pressure at the well (Pa)
*Q*	total amount of injected heat (J)
*q*	heat injection rate (W/m of well)
*Q*_P_	gas production rate (ST m^3^/day/m of well)
*Q*_W_	water production rate (kg/day/m of well)
*r*	radius (m)
*r*_W_	wellbore radius (m)
*R*_GW_	gas-to-water ratio (ST m^3^ of CH_4_/m^3^ of H_2_O)
*S*	phase saturation
*t*	time (days)
*T*	temperature (°C)
*T*_0_	permafrost ground temperature (°C)
*T*_B_	initial temperature at the base of HBL (°C)
*V*_P_	cumulative volume of produced CH_4_ (ST m^3^)
*W*	pump work (J)
x,y,z	cartesian coordinates (m)
Δ*H*_C_	combustion enthalpy of produced methane (J)
Δ*l*_I-P_	horizontal distance between injector and producer (m)
Δ*l*_P-P_	vertical distance between two producers (m)
*Φ*	porosity
*η*	energy ratio
*χ*	hydrate dissociation percentage
*λ*	van Genuchten exponent—Table 1
**Subscripts and Superscripts**	
0	denotes initial state
A	aqueous phase
B	base of HBL
cap	capillary
G	gas phase
H	solid hydrate phase
I	solid ice phase
irA	irreducible aqueous phase
irG	irreducible gas
*n*	permeability reduction exponent—Table 1
*n*_G_	gas permeability reduction exponent—Table 1
W	well
